# Impact of Postmastectomy Radiotherapy on Locoregional Control and Disease-Free Survival in Patients with Breast Cancer Treated with Neoadjuvant Chemotherapy

**DOI:** 10.1155/2021/6632635

**Published:** 2021-01-24

**Authors:** Yanyu Zhang, Yaotian Zhang, Zhuang Liu, Zilan Qin, Yubing Li, Jiaming Zhao, Xinchi Ma, Qiankun Yang, Ning Han, Xue Zeng, Hong Guo, Na Zhang

**Affiliations:** ^1^Department of Radiation Oncology, Cancer Hospital of China Medical University, Liaoning Cancer Hospital and Institute, Shenyang Liaoning 110042, China; ^2^Department of Bone and Soft Tissue Tumour Surgery, Cancer Hospital of China Medical University, Liaoning Cancer Hospital and Institute, Shenyang 110042, China

## Abstract

**Background:**

The impact of postmastectomy radiotherapy (PMRT) in patients receiving neoadjuvant chemotherapy (NAC) is unclear. The purpose of this study is to identify the patients who may benefit from PMRT.

**Methods:**

We retrospectively analysed patients with clinical stage II-III breast cancer who underwent NAC and modified radical mastectomy at our centre from 2007 to 2015. We investigated the relationship amongst locoregional recurrence rate (LRR), disease-free survival (DFS), and clinical pathological characters.

**Results:**

A total of 554 patients were analysed in this study. The median follow-up time was 65 months. Amongst the patients, 58 (10.5%) had locoregional recurrence, 138 (24.9%) had distant metastasis, and 72 (13.0%) patients died. The 5-year cumulative incidence of LRR and DFS was 9.2% and 74.2%, respectively. A total of 399 (72%) patients received PMRT and 155 (28%) did not. The 5-year LRR of the patients with PMRT (7.3% vs. 14.1%, *P*=0.01) decreased significantly. We found that PMRT was an independent prognostic factor of LRR and DFS. Patients with the persistent involvement of 1–3 lymph nodes (ypN1) and more than 4 positive lymph nodes (ypN2-3) had a better outcome after PMRT than those without. However, the LRR and DFS of patients with negative lymph nodes at the time of surgery (ypN0) and who received PMRT showed no significant benefits. Amongst all patients with the three molecular subtypes of breast cancer, patients with triple-negative breast cancer had the highest pathological complete response rate but the worst prognosis (*P*=0.001).

**Conclusion:**

Results showed that PMRT significantly reduced the LRR of patients with clinical stage II-III breast cancer after receiving NAC and mastectomy. YpN0 patients derived no local control or survival benefit after receiving PMRT, whereas those with ypN1 and ypN2-3 could obviously benefit from PMRT.

## 1. Introduction

Neoadjuvant chemotherapy (NAC) is currently widely used in patients with locally advanced breast cancer to transform nonoperable cases into operable ones, thus improving breast preservation rates and reflecting the sensitivity of tumour cells to systemic therapy [1–3]. A large number of studies have shown that postmastectomy radiotherapy (PMRT) can significantly reduce the locoregional recurrence rate (LRR) of patients with stage II-III breast cancer and increase overall survival (OS) [4, 5]. However, clear data regarding the role of adjuvant radiotherapy in the context of NAC remain lacking [4, 6–9].

A number of retrospective studies have shown that PMRT can reduce the LRR of patients with NAC [10–13], but due to the change in tumour characters after NAC, the optimal indication for PMRT for the management of patients treated with NAC remains controversial. An analysis of experiments on the two prospective neoadjuvants B-18 and B-27 by the National Surgical Adjuvant Breast and Bowel Project (NSABP) in the United States suggested that tumour response and pathological lymph node status are independent prognostic factors of LRR [14].

This study mainly aimed to explore the independent prognostic factors affecting the LRR and disease-free survival (DFS) of patients with clinical stage II-III breast cancer undergoing NAC and mastectomy and to identify with increased accuracy the patients that should receive PMRT following NAC.

### 1.1. Patients and Methods

This study retrospectively analysed the patients with clinical stage II-III breast cancer at our centre from 2007 to 2015. The patients who received NAC and modified radical mastectomy (MRM) after fine-needle aspiration biopsy and had breast cancer diagnosis and complete treatment data were analysed. Patients with inflammatory breast cancer, male breast cancer, simultaneous bilateral breast cancer, or a history of other malignant tumours were excluded. Patients with the metastasis of supraclavicular/internal breast lymph nodes or distant metastases before NAC were also excluded.

Breast tumour size and axillary lymph node status were evaluated via physical examination and imaging examination and staged on the basis of the seventh edition of the American Joint Committee on Cancer TNM staging classification. Hormone receptor status was determined on the basis of reference pathology before treatment. Estrogen receptor (ER) and progesterone receptor (PR) positivity was defined as positive immunohistochemical staining in ≥1% of tumour cells. Human epidermal growth factor receptor (HER-2) positivity was defined as the immunohistochemical detection of 3+ or 2+ with amplification via fluorescent in situ hybridization. Triple-negative breast cancer (TNBC) was defined as negative for ER, PR, and HER-2. Pathological complete response (pCR) was defined as the absence of residual invasive tumours in primary and axillary lesions.

LRR was the first endpoint, and DFS was the second endpoint. LRR was defined as recurrent disease in the chest wall and/or the ipsilateral internal mammary, axillary, or supraclavicular nodes. DFS was defined as the absence of locoregional recurrence and distant metastasis (including the metastasis of the contralateral breast, bone, liver, lung, brain, or other organs) or death from any cause and was diagnosed by pathological or imaging examinations, including computed tomography, ultrasound, magnetic resonance imaging (MRI), and bone scans.

### 1.2. Treatment

All patients received NAC. Every two cycles of chemotherapy, the size of the tumour was evaluated through ultrasound examination, and efficacy evaluation was performed in accordance with the response evaluation criteria for solid tumours (RECIST 1.1). NAC plus targeted therapy was feasible for HER-2 positive (HER-2+) patients. All patients underwent MRM for breast cancer. The patients received adjuvant chemotherapy, radiotherapy, or endocrine therapy after NAC in accordance with the clinical pathological characteristics of the tumour. HER-2+ patients continued to use trastuzumab after surgery for a total duration of 1 year. Radiotherapy included conventional external radiation, two-dimensional radiation, three-dimensional conformal radiation therapy or intensity-modulated radiation therapy. The clinician formulated a plan and guided the treatment in accordance with the clinical pathological characteristics of the tumour.

### 1.3. Statistical Analysis

Collected patient information was subjected to statistical analysis by using SPSS 25.0 software and GraphPad Prism8. Descriptive statistics was used to describe each variable. The Kaplan–Meier method was used to describe the LRR and DFS survival curves. The log-rank test was used to compare the survival curves. *P* < 0.05 indicated that the difference was statistically significant. The Cox risk proportional model was applied to evaluate the effect of variables that were related to LRR and DFS, and the independent prognostic factors of LRR and DFS were clarified. Hazard ratios (HR) and 95% confidence intervals (CI) were given. A two-sided *P* value of 0.05 was used as the alpha error for the consideration of statistical significance.

## 2. Results

### 2.1. Baseline Characteristics of This Cohort

A total of 554 patients were analysed in this study. The median follow-up time was 65 months from diagnosis. The clinical and pathological characteristics of the patients are introduced in detail in Table 1. The median age at diagnosis was 51 years (range: 22–78 years). Amongst the patients, 394 (71.1%) and 160 (28.9%) were in clinical stages II and III, respectively. A total of 75% of the patients received anthracycline and paclitaxel chemotherapy regimens, and 7% of the patients received platinum-containing chemotherapy regimens.

A total of 472 (85.1%) patients achieved complete response (CR) or partial response (PR) after NAC, and 38 (6.9%) reached pCR after surgery. The numbers of patients who had negative lymph nodes at the time of surgery (ypN0), persistent involvement of 1–3 lymph nodes (ypN1), and more than 4 positive lymph nodes (ypN2-3) were 177 (32.0%), 192 (34.6%), and 185 (33.4%), respectively. A total of 453 (81.6%) patients received adjuvant chemotherapy after surgery, and 284 (83.2%) of of hormone receptor positive (HR+) patients received endocrine therapy. A total of 399 (72%) of 554 patients received PMRT to the chest wall and/or the regional lymph nodes. No statistical difference in age, menstrual status, histological grade, pathological type, hormone receptor status, and HER-2 status was observed between the PMRT group and the non-PMRT group. Patients who received PMRT had significantly more advanced tumour and nodal stages before and after NAC than those who did not receive PMRT. The rates of clinical stage II and stage III in both groups were 65.9% vs. 84.5% (*P* ≤ 0.001) and 34.09% vs. 15.48% (*P* ≤ 0.001). In the non-PMRT group, 60.7% patients were ypN0, whereas ypN0 patients accounted for only 20.8% of the PMRT group.

### 2.2. Local Recurrence Pattern and Independent Prognostic Factors of LRR and DFS

A total of 58 (10.5%) patients experienced local recurrence, including 28 (5.1%) patients with ipsilateral chest wall recurrence, 7 (1.3%) patients with axillary nodal recurrence, 14 (2.5 %) patients with ipsilateral supraclavicular recurrence, and 9 (1.6%) with simultaneous recurrence at two or more positions.

**Figure 1:**
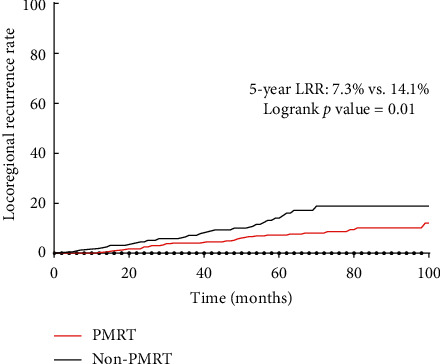
Kaplan–Meier plot for the cumulative incidence of locoregional recurrence.

**Figure 2:**
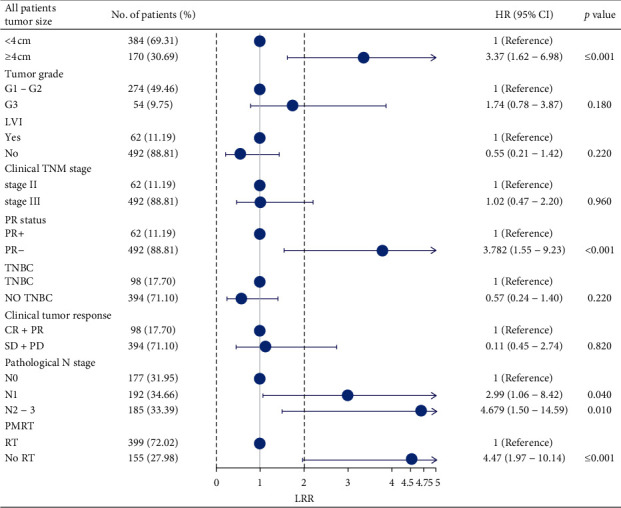
Results of the multivariate Cox regression analysis of LRR. LRR: locoregional recurrence; PMRT: postmastectomy radiation therapy; TNBC: triple-negative breast cancer; LVI: lymphatic vascular infiltration; PR: progesterone receptor; TNBC: triple-negative breast cancer; pCR: pathological complete response; CR: complete response; PR: partial response; SD: stable disease; PD: progressive disease; HR: hazard ratio; CI: confidence interval.

A total of 34 (8.2%) patients in the PMRT group and 24 (15.5%) in the non-PMRT group had locoregional recurrence. The 5-year LRR of the PMRT group was significantly lower than that of the non-PMRT group (7.3% vs. 14.1%, *P*=0.01) as shown in Figure 1. Univariate analysis showed that the factors associated with LRR included (Table 2) clinical tumour size, histological grade, clinical stage, LVI, PR, ypN stage, TNBC, tumour chemotherapy response, and PMRT. Incorporating these factors into the Cox risk proportional model revealed that patients with cT ≥4 cm, PR negativity, ypN 1, and ypN 2–3 and without PMRT had poor LRR (Figure 2). The LRR of patients without PMRT was 4.47 times that of patients with PMRT (CI 1.97–10.14, *P* ≤ 0.01), whereas the LRR of ypN2-3 patients was 4.68 times that of ypN0 patients.

**Figure 3:**
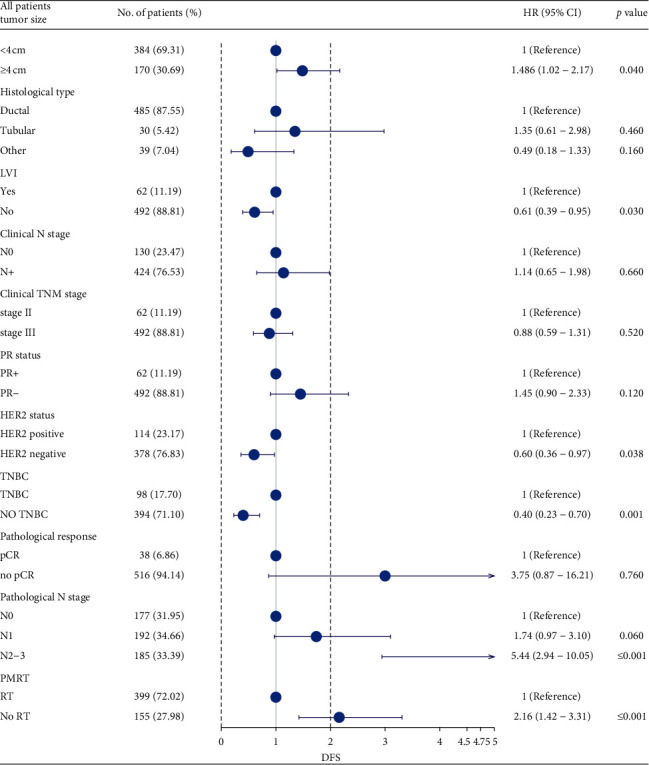
Results of the multivariate Cox regression analysis of DFS. DFS: disease-free survival; PMRT: postmastectomy radiation therapy; TNBC: triple-negative breast cancer; LVI: lymphatic vascular infiltration; ER: estrogen receptor; PR: progesterone receptor; HER-2: human epidermal growth factor receptor; TNBC: triple-negative breast cancer; pCR: pathological complete response; HR: hazard ratio; CI: confidence interval.

A total of 138 (25.0%) patients in the whole group had distant metastasis (including 10 patients with contralateral breast metastasis). DFS did not obviously differ between the PMRT group and non-PMRT group (74% vs. 74.8%, *P*=0.99). Univariate analysis revealed that the factors affecting DFS included (Table 2) cT, pathological type, clinical stage, cN, lymph-vascular infiltration (LVI), PR, HER-2, ypN, and TNBC. Considering that some studies have shown that PMRT can affect the DFS of patients, PMRT and the related factors of the above univariate analysis were included in the multivariate analysis simultaneously. The independent prognostic factors of DFS were cT, PR, LVI, TNBC, HER-2, ypN, and PMRT (Figure 3).

### 2.3. ypN0, ypN1, and ypN2-3

The number of positive lymph nodes is an important variable that affects LRR and DFS. The 5-year LRR of ypN0 patients was significantly lower than that of ypN + patients (5.3% vs. 11.1%, *P*=0.01). LRR gradually increased following the increase in the number of positive lymph nodes. The 5-year LRRs of the ypN0, ypN1, and ypN2-3 cohorts were 5.3%, 7.8%, and 14.7%, respectively.

**Figure 4:**
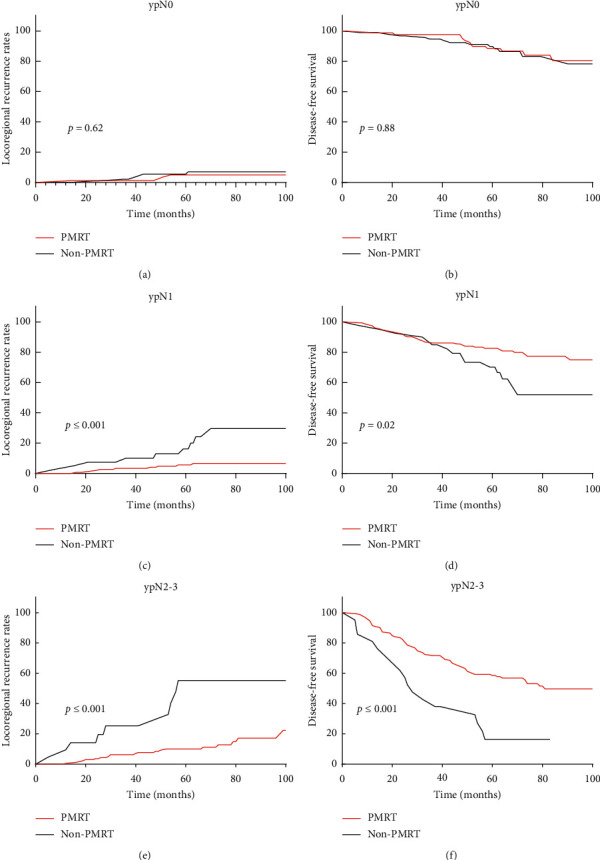
Locoregional recurrence and disease-free survival of patients with breast cancer with or without PMRT in the (a) LRR of ypN0, (b) DFS of ypN0, (c) LRR of ypN1, (d) DFS of ypN1, (e) LRR of ypN2-3, and (f) DFS of ypN2-3.

For patients with ypN0, the 5-year LRR was not significantly different between the PMRT group and non-PMRT group (*P*=0.62, Figure 4(a)). 4 (4.8%) and 6 (6.3%) of the patients in the PMRT and non-PMRT groups experienced locoregional recurrence, respectively. DFS did not differ between the two groups (*P*=0.88) (Figure 4(b)).

However, in the ypN1 and ypN2-3 cohorts, the 5-year LRR rates were significantly decreased in the patients with PMRT (ypN1: *P* ≤ 0.001, ypN2-3: *P* ≤ 0.001) (Figures 4(c) and 4(e)). Moreover, the DFS of these patients improved (ypN1: *P*=0.02; ypN2-3: *P* ≤ 0.001) (Figures 4(d) and 4(f)).

### 2.4. Molecular Subtypes

**Figure 5:**
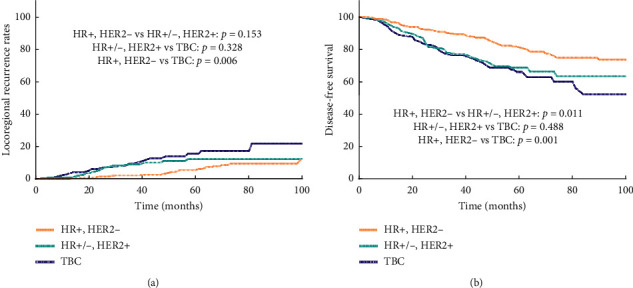
(a) Locoregional recurrence of three molecular subtypes. (b) Disease-free survival of the three molecular subtypes.

**Figure 6:**
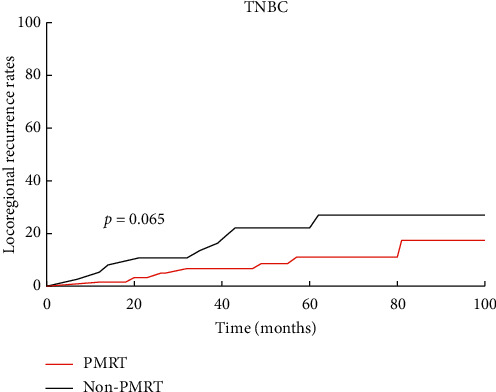
Locoregional recurrence of TNBC patients with or without PMRT.

Survival curves showed significant differences in LRR and DFS amongst the three subtypes (HR+ and HER-2−, HER-2+, and TNBC) (Figure 5). The 5-year LRR of patients with TNBC was significantly higher than that of HR+ and HER-2− patients (15.3% vs. 5.6%, *P*=0.006) (Figure 5(a)), whereas the 5-year DFS of TNBC and HER-2+ patients were worse than those of HR+ and HER-2− patients (81.3% vs. 66.3%, *P*=0.001; 81.3% vs. 68.8%, *P*=0.011) (Figure 5(b)). No significant difference was observed in the LRR and DFS between the PMRT group and the non-PMRT group in the three subtypes. However, the survival curve of TNBC patients indicated that PMRT was beneficial (*P*=0.065) (Figure 6).

A clear relationship was observed between tumour response and subtypes. The pCR rates of HR+, HER-2+, and TNBC patients were 3.6%, 9.6%, and 14.3%, respectively. TBC and HER + patients were more likely to achieve pCR than HR + patients after receiving NAC (Table 3: HER-2+: OR = 4.40, 95% CI: 1.89–10.27, *P*=0.001; TNBC: OR = 2.82, 95% CI: 1.16–6.84, *P*=0.02).

## 3. Discussion

NAC has been recently widely used in patients with clinical stage II or III breast cancer. Although NAC has improved the surgical outcomes for many patients with breast cancer, the risk of relapse remains high especially for patients with locally advanced breast cancer. However, the indication for PMRT in patients treated with NAC remains controversial given the lack of prospective evidence.

Multiple studies have shown that pCR is an important prognostic indicator for survival [15–17]. NSABP B-18/27 analysis confirmed that, amongst patients with clinical stage I-II disease, pCR patients had better DFS and OS than non-pCR patients. McGuire believed that patients with stage I-II pCR do not need radiotherapy. However, even for patients with stage III breast cancer who achieved pCR after NAC, PMRT can still reduce LRR by 26% and significantly improve OS [14]. A retrospective study obtained a similar conclusion that PMRT can still reduce the LRR of patients with clinical III-IV disease and pCR in 10 years (3% vs. 33%; *P*=0.006) but does not benefit patients with clinical stage I-II disease [10].

In our study, 83.23% of 554 patients exhibited tumour shrinkage of more than 30% after NAC, 9.9% of patients experienced the complete disappearance of primary breast tumours, and 6.8% of patients achieved pCR because only 18% of patients underwent full-course NAC. Therefore, the proportion of postoperative pCR patients in our study was lower than that in other studies.

The number of positive lymph nodes is an important prognostic indicator [18, 19]. Some studies thought that the initial lymph node status before NAC and postoperative lymph node status should be referenced to consider whether the patient should accept PMRT [20, 21]. However, some experts disagree with this view and believe that patients with N0 can be exempted from radiotherapy [10, 22]. A 2016 National Cancer Database study involving 10 283 patients with clinical T1-3N1M0 breast cancer (ypN2-3) identified PMRT as an independent prognostic factor of improving OS. After receiving PMRT, the OS of patients in each layer of ypN (ypN0, ypN1, and ypN2-3) improved [20].

Another study showed that the risk of regional recurrence, distant metastasis, and death did not increase in 56 (41.8%) out of 134 ypN0 patients without PMRT compared with that of 78 (58.2%) patients who received PMRT [18]. Shim and Kantor et al. similarly concluded that PMRT may be unnecessary in patients with ypN0 regardless of clinical stage [23, 24]. A study explored the benefit of PMRT in ypN0 patients after NAC in accordance with molecular subtypes. A total of 189 patients were included in this analysis, and the effects of PMRT on locoregional control, DFS, and OS were evaluated. However, PMRT provides no additional survival benefits for any molecular subtype [25].

A number of retrospective studies have shown that sentinel lymph node biopsy (SLNB) after NAC can prevent patients with negative axillary lymph nodes from undergoing unnecessary axillary lymph node dissection (ALND) [26–28]. However, the false negative of SNLB can lead to incorrect lymph node staging and affect the formulation of comprehensive treatment plans after surgery. Therefore, the patients analysed in this study all underwent ALND. Whether SNLB can replace ALND still needs to be further verified in the prospective study Alliance A011202.

Patients with lymph node positivity after surgery are strongly recommended for PMRT [29, 30]. However, experts have not reached a consensus on whether ypN0 patients require PMRT and still expect confirmation from the prospective randomised controlled trial RTOG1304/NSABP B51.

This study has numerous deficiencies given the limitations of retrospective research. The baseline characteristics of the patients in the two groups were different. Nearly 90% of the patients' lymph node status before neoadjuvant therapy was based on clinical examination or ultrasound, mammography, or MRI. Moreover, only 10% patients underwent lymph node fine-needle aspiration biopsy. Thus, a certain error existed in the judgment of preoperative lymph node status. Standard MRM involves the removal of the affected breast and parallel ALND with the number of lymph nodes removed ≥10. In our study, we included 15 patients who underwent ALND but had only 5–9 lymph node dissections. The patients received different treatments during different periods with the change in NAC regimen, chemotherapy cycle, and radiotherapy technology. In addition, radiotherapy dose and irradiation field details were missing. Patients with PMRT cannot be compared. Patients with breast cancer generally have a good prognosis. Given that the median follow-up time of this study was 65 months, observation must be extended to further evaluate the prognosis of patients.

## 4. Conclusion

In this retrospective study, we found that patients with clinical stage II-III breast cancer receiving NAC exhibited reduced LRR after PMRT and those with ypN1 and ypN2-3 showed significantly reduced LRR and improved DFS after receiving PMRT. Patients with pCR and ypN0 did not present increased LRR and DFS after exemption from PMRT. Prospective trials are expected to verify whether radiotherapy can be omitted for these patients.

## Figures and Tables

**Table 1:** Clinical pathological characteristics of all patients (*N* = 554).

Variable	All		RT		No RT		*P* value
	*N* = 554	%	*N* = 399	%	*N* = 155	%	
Age (years)							0.980
<50	239	43.1	172	43.10	67	43.20	
≥50	315	56.9	227	56.90	88	56.80	
Menopausal status							0.609
Perimenopausal	255	46.0	184	46.11	71	45.81	
Postmenopausal	265	47.8	193	48.37	72	46.45	
Not known	34	6.1	22	5.51	12	7.74	
Side of primary tumour							0.812
Left	294	53.1	213	53.38	81	52.26	
Right	260	46.9	186	46.62	74	47.74	
Tumour size, cm							≤0.001
<4	384	69.3	258	64.67	126	81.29	
≥1	170	30.7	141	35.33	29	18.71	
Tumour grade							0.445
G1-G2	274	49.5	194	48.62	80	51.61	
G3	54	9.8	41	20.28	13	8.39	
Not known	226	40.8	164	41.10	62	40	
Histological type							0.449
Ductal	485	87.6	345	86.47	140	90.32	
Tubular	30	5.4	24	6.02	6	3.87	
Others	39	7.0	30	7.52	9	5.81	
ER status							0.618
ER+	336	60.6	245	61.40	91	58.70	
ER−	217	39.2	154	38.60	63	40.60	
PR status							0.216
PR+	264	47.7	197	49.40	67	43.23	
PR−	289	52.2	202	50.60	87	56.13	
HER-2 status							0.447
HER-2 positive	114	20.6	85	21.30	29	18.71	
HER-2 negative	378	68.2	268	67.20	110	80.00	
Not recorded	62	11.2	46	11.50	16	10.30	
TBC							0.020
Yes	98	17.7	61	15.30	37	23.90	
No	394	71.1	292	73.20	102	65.80	
Clinical T stage							≤0.001
T1-T2	438	79.1	297	74.44	141	90.97	
T3-T4	116	20.9	102	25.57	14	9.03	
Clinical N stage							≤0.001
N0	130	23.5	69	17.30	61	39.35	
N1	339	61.2	260	65.16	79	50.97	
N2	85	15.3	70	17.54	15	9.68	
Clinical TNM stage							≤0.001
II	394	71.1	263	65.91	131	84.52	
III	160	28.9	136	34.09	24	15.48	
Clinical tumour response							0.415
CR + PR	472	85.2	343	85.96	129	83.23	
SD + PD	82	14.8	56	14.04	26	16.77	
Pathological response							≤0.001
pCR	38	6.9	15	3.76	23	14.84	
No pCR	516	94.1	384	96.24	132	85.16	
LVI							0.315
Yes	62	11.2	48	12.03	14	9.03	
No	492	88.8	351	87.97	141	90.97	
Pathological T stage							≤0.001
T0/tis	55	9.9	29	7.27	26	16.77	
T1	279	50.4	199	49.87	80	51.61	
T2–T4	220	39.7	171	42.86	49	31.61	
Pathological N stage							≤0.001
N0	177	31.0	83	20.80	94	60.65	
N1	192	34.7	152	38.10	40	25.81	
N2-3	185	33.3	164	41.10	21	13.55	
Pathological stage							≤0.001
0	38	6.9	15	3.76	23	14.84	
I	94	16.0	44	11.03	50	32.26	
II	228	41.2	168	42.11	60	38.71	
III	194	35.0	172	43.11	22	14.19	

PMRT, postmastectomy radiation therapy; ER, estrogen receptor; PR, progesterone receptor; HER-2, human epidermal growth factor receptor; TNBC, triple-negative breast cancer; pCR, pathological complete response; CR, complete response; PR, partial response; SD, stable disease; PD, progress disease; LVI, lymphatic vascular infiltration; HR, hazard ratio.

**Table 2:** Results of univariate analysis for locoregional recurrence and disease-free survival.

Variable	LRR		DFS	
	HR (95% CI)	*P* value	HR (95% CI)	*P* value
Age (years)		0.965		0.173
<50	1 (ref)		1 (ref)	
≥50	0.988 (0.589–1.659)		1.243 (0.909–1.698)	
Tumour size, cm		0.01		≤0.001
<4	1 (ref)		1 (ref)	
≥r	2.355 (1.404–3.952)		1.758 (1.286–2.403)	
Tumour grade		0.015		0.488
G1-G2	1 (ref)		1 (ref)	
G3	2.377 (1.182–4.778)		1.203 (0.713–2.031）	
Histological type		0.806		0.035
Ductal	1 (ref)		1 (ref)	
Tubular	1.097 (0.0396–3.039)		1.132 (0.613–2.089）	
Others	0.692 (0.216–2.217)		0.302 (0.112–0.814）	
LVI		0.093		≤0.001
Yes	1 (ref)		1 (ref)	
No	0.557 (0.282–1.103)		0.486 (0.327–0.723）	
Clinical N stage		0.461		0.007
N0	1 (ref)		1 (ref)	
N1	1.401 (0.733–2.676)		1.979 (1.292–3.031）	
N2	0.97 (0.382–2.465)		1.624 (0.937–2.812）	
Clinical TNM stage		0.044		0.002
Stage II	1 (ref)		1 (ref)	
Stage III	1.718 (1.015–2.907)		1.651 (1.205–2.260）	
ER status		0.405		0.901
ER+	1 (ref)		1 (ref)	
ER−	1.249 (0.743–2.101)		1.020 (0.746–1.395）	
PR status		0.002		0.033
PR+	1 (ref)		1 (ref)	
PR−	2.428 (1.379–4.276)		1.400 (1.027–1.909）	
HER-2 status		0.483		0.094
HER-2 positive	1 (ref)		1 (ref)	
HER-2 negative	0.797 (0.424–1.500)		0.726 (0.499–1.056）	
TNBC		0.017		0.006
TNBC	1 (ref)		1 (ref)	
No TNBC	0.485 (0.268–0.878)		0.592 (0.405–0.863)	
Clinical tumour response		0.012		0.273
CR + PR	1 (ref)		1 (ref)	
SD + PD	2.126 (1.180–3.831)		1.255 (0.836–1.886）	
Pathological response		0.162		0.010
pCR	1 (ref)		1 (ref)	
No pCR	4.099 (0.567–29.615)		6.262 (1.553–25.256）	
Pathological N stage		≤0.001		≤0.001
N0	1 (ref)		1 (ref)	
N1	1.712 (0.79–3.711)		1.753 (1.085–2.832）	
N2	2.547 (1.166–5.565)		3.554 (2.231–5.662）	
N3	5.644 (2.472–12.882)		7.249 (4.371–12.022）	
PMRT		0.013		0.993
RT	1 (ref)		1 (ref)	
No RT	1.935 (1.147–3.266）		0.999 (0.708–1.407）	
Anti-HER-2 therapy		0.629		0.983
Yes	1 (ref)		1 (ref)	
	0.751 (0.235–2.401）		1.007 (0.546–1.856）	
Adjuvant chemotherapy		0.184		0.524
Yes	1 (ref)		1 (ref)	
No	1.238 (0.903–1.695）		1.083 (0.847–1.384）	
Endocrinotherapy		0.218		0.121
Yes	1 (ref)		1 (ref)	
No	1.119 (0.935–1.339）		1.093 (0.977–1.223）	

LRR, locoregional recurrence rates; DFS, disease-free survival; HR, hazard ratio; CI, confidence interval; ER, estrogen receptor; PR, progesterone receptor; HER-2, human epidermal growth factor receptor; TNBC, triple-negative breast cancer; pCR, pathological complete response; CR, complete response; PR, partial response; SD, stable disease; PD, progress disease.

**Table 3:** Relationship between pCR rates and molecular subtypes.

Molecular	pCR rates (%)	OR (95% CI)	*P*
HR+HER-2−	3.60	1 (ref)	—
HR+/HR−, HER-2+	9.60	2.82 (1.16–6.84)	0.02
TBC	14.30	4.40 (1.89–10.27)	0.001

TNBC: triple-negative breast cancer; HR: hormone receptor; HER-2 human epidermal growth factor receptor; TNBC: triple-negative breast cancer; pCR: pathological complete response; HR: hazard ratio; CI: confidence interval.

## Data Availability

The raw/processed data required to reproduce these findings cannot be shared at this time as the data also form part of an ongoing study.
